# Beyond the Aorta: Incidental Atrial Septal Defect in a Patient With Marfan Syndrome and Severe Aortic Dilation

**DOI:** 10.7759/cureus.104956

**Published:** 2026-03-09

**Authors:** Freddy J Medina Santos, Jaime E Pérez Figueroa, Rodrigo A Bonilla Figueroa, Kevin J Acevedo Gómez, Ana K Garro Almendaro, Ricardo A Cruz Villalobos, José F García Rodríguez

**Affiliations:** 1 Cardiology, Centro Médico Nacional 20 de Noviembre, Mexico City, MEX; 2 Echocardiography, Centro Médico Nacional 20 de Noviembre, Mexico City, MEX

**Keywords:** aortic aneurysm, aortic regurgitation, atrial septal defect, bentall procedure, fibrillin-1, marfan syndrome

## Abstract

Marfan syndrome (MFS) is an autosomal dominant connective tissue disorder caused by mutations in the FBN1 gene, which carries a high risk of cardiovascular morbidity and mortality. We present the case of a 31-year-old man with a severe Marfanoid phenotype who was admitted with decompensated heart failure, septic shock, and renal failure. After stabilization, multimodal evaluation revealed a severely dilated aortic root and severe aortic regurgitation, along with an incidental 11 mm ostium secundum atrial septal defect (ASD). This unusual combination caused critical biventricular overload. The patient underwent a successful Bentall-Bono procedure and closure of the ASD. This case underscores the importance of a thorough physical examination and comprehensive diagnosis for successful surgery in patients with complex connective tissue disorders.

## Introduction

Marfan syndrome (MFS) is a hereditary genetic disorder with an autosomal dominant inheritance pattern that affects connective tissue. It has a prevalence of two to three per 10,000 individuals in the general population [1]. It is characterized by multisystem involvement, with predominant involvement of the cardiovascular, musculoskeletal, and ocular systems. Its etiology lies in a mutation of the FBN1 gene on chromosome 15, which encodes the FBN1 protein. FBN1 is essential for maintaining the integrity of extracellular matrix microfibrils, which are critical for the structural integrity and elasticity of tissues [2].

Among all the clinical manifestations, cardiovascular ones are typically the most critical and those that determine the long-term prognosis [1]. Alterations in the extracellular matrix lead to progressive weakening of the aortic media, known as cystic medial degeneration [3]. This results in dilation of the aortic root and ascending aorta, predisposing patients to a higher risk of aortic dissection and rupture, as well as failure of aortic valve coaptation, leading to aortic insufficiency. This insufficiency can be progressive and severe, compromising the patient's clinical status and prognosis [1].

Severe aortic insufficiency causes chronic volume and pressure overload of the left ventricle, leading to dilation and eventual dysfunction and, ultimately, heart failure [1].

The diagnosis of MFS has evolved towards the revised Ghent nosology, which prioritizes aortic root dilation (Z-score ≥ 2) and ectopia lentis as cardinal features [4]. In the absence of these, the diagnosis relies on a formal systemic score. This scoring system quantifies diverse clinical manifestations such as skeletal deformities, ligamentous laxity, and ocular abnormalities, where a total of seven or more points indicates definitive systemic involvement [4]. This standardized approach allows for early detection and risk stratification, which are crucial pillars in the management of these patients [5].

The therapeutic strategy combines lifestyle modifications, optimal medical treatment, and timely surgical intervention [5]. Medical treatment prioritizes controlling blood pressure and heart rate to reduce stress on the aorta, thereby decreasing the rate of aortic dilation and dissection. This requires the use of medications such as beta-blockers and/or angiotensin receptor antagonists. However, the definitive treatment is surgical, which will depend on the aortic diameter, the rate of growth, and the presence of symptomatic valvular insufficiency. Surgical correction, even in the presence of other congenital heart defects, is a fundamental strategy for improving survival and quality of life in patients with MFS [5,6].

## Case presentation

We present the case of a 31-year-old man with a prior clinical suspicion of MFS and a family history of sudden death. The patient was initially admitted to a secondary-care center for decompensated heart failure, septic shock of pulmonary origin, and acute kidney injury requiring a single session of renal replacement therapy. He completed an empiric antibiotic regimen with meropenem and vancomycin. Upon transfer to our institution 20 days after the initial admission, the patient showed no clinical or laboratory evidence of systemic inflammatory response syndrome. At that time, renal function had normalized, with a creatinine level of 0.74 mg/dL and adequate urinary output.

**Figure 1:**
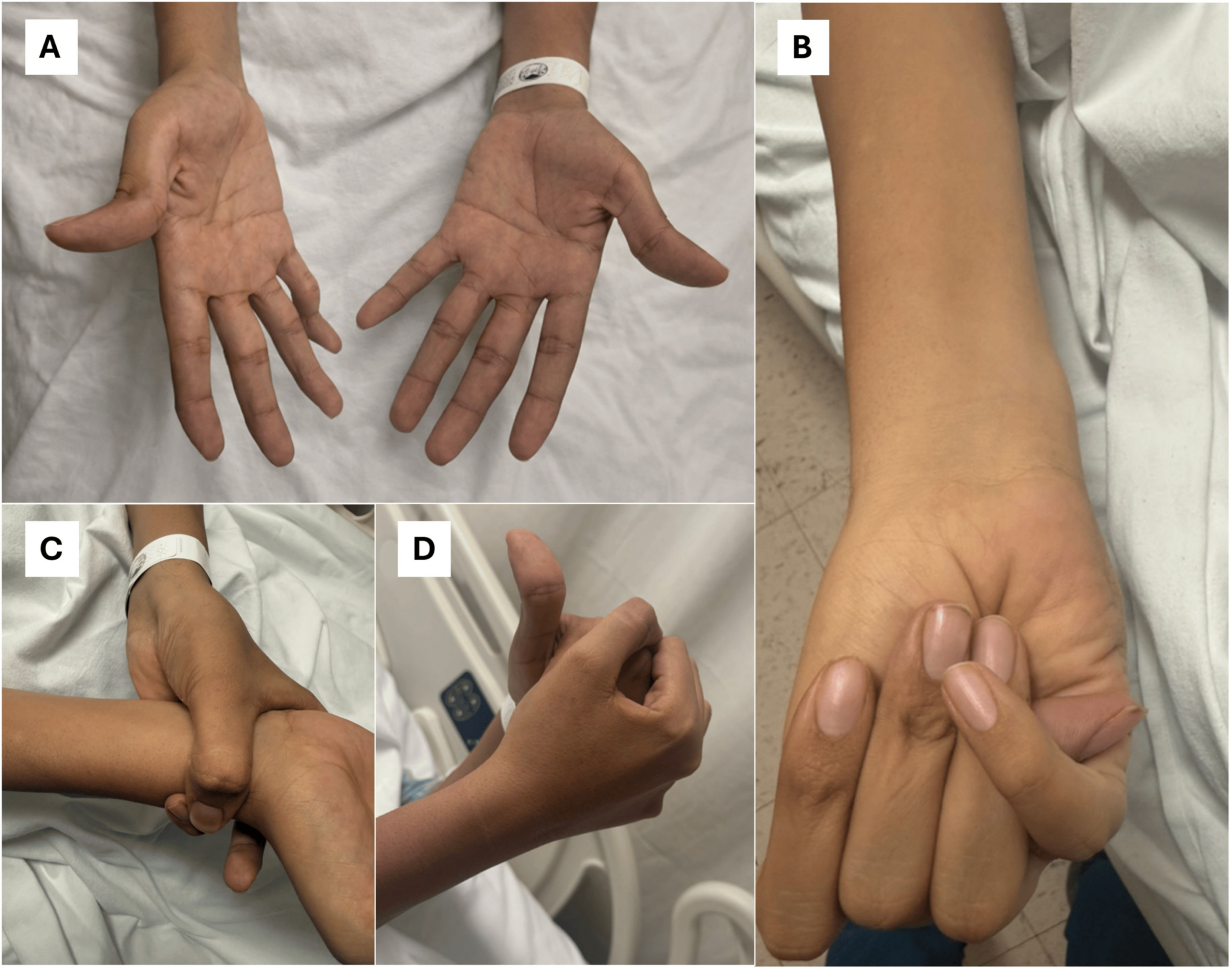
Clinical manifestations of the Marfanoid phenotype in the upper extremities A. Evident arachnodactyly with disproportionate lengthening of the phalanges; B. Positive Steinberg sign (thumb sign): the distal phalanx of the adducted thumb extends beyond the ulnar border of the clenched fist; C. Positive Walker-Murdoch sign (wrist sign): the thumb and fifth finger overlap when encircling the contralateral wrist; D. Extreme ligamentous laxity and severe arachnodactyly, evidenced by the ability to encompass the contralateral hand fully.

During the clinical evaluation of the upper extremities, classic findings of MFS were documented. The patient presented with arachnodactyly and a positive Steinberg sign (thumb), where the distal phalanx of the adducted thumb clearly protruded from the ulnar border of the closed hand. Likewise, the Walker-Murdoch sign (wrist) was positive, demonstrating complete overlap between the thumb and the fifth finger when encircling the contralateral wrist. Extreme ligamentous laxity and disproportionate finger length were also observed, allowing the patient to almost completely cover one hand with the other in a fist, reflecting the severity of the generalized connective tissue disease (Figure 1). These findings allowed us to calculate a systemic score of 8 points according to the revised Ghent nosology [4], providing definitive clinical confirmation of MFS in the absence of previous genetic testing (Table 1).

**Table 1:** Systemic score calculation based on the revised Ghent nosology This table summarizes the systemic score calculation for the patient according to the diagnostic criteria established in the revised Ghent nosology for the Marfan syndrome [4]. A score ≥ 7 points is considered indicative of systemic involvement. In this case, the patient achieved a total score of 8 points, providing definitive clinical evidence of the syndrome. US/LS: upper segment/lower segment ratio

Systemic Feature	Points	Patient Findings	Points Awarded
Wrist and thumb sign	3	Positive (both)	3
Pectus carinatum deformity	2	Not present	0
Pectus excavatum or chest asymmetry	1	Not present	0
Hindfoot deformity/plain pes planus	2/1	Present (Plain pes planus)	1
Pneumothorax	2	Not present	0
Dural ectasia	2	Not evaluated	0
Protrusio acetabuli	2	Not evaluated	0
Reduced US/LS and increased arm/height and no severe scoliosis	1	Present	1
Scoliosis or thoracolumbar kyphosis	1	Not present	0
Reduced elbow extension	1	Present	1
Facial features (3/5) (dolichocephaly, enophthalmos, downslanting palpebral fissures, malar hypoplasia, retrognathia)	1	Present	1
Skin striae	1	Not present	0
Myopia > 3 diopters	1	Present	1
Mitral valve prolapse (all types)	1	Not present	0
MAXIMUM TOTAL	20	PATIENT TOTAL	8

Given the presentation of fever, sepsis, and severe aortic regurgitation, infective endocarditis (IE) was the primary differential diagnosis. IE was excluded as the patient did not meet the Modified Duke Criteria [7]: serial blood cultures were negative, and both transthoracic echocardiography (TTE) and computed tomography (CT) showed no evidence of vegetations, abscesses, or valvular perforations. Transesophageal echocardiography (TEE) and fluorodeoxyglucose (FDG)-PET were not performed due to patient intolerance and resource availability, respectively. Furthermore, no embolic or immunologic phenomena were identified.

Following hemodynamic stabilization, a TTE was performed. The aortic root analysis revealed an annulus of 28 mm (indexed: 12.7 mm/m²) and sinuses of Valsalva of 63 mm (indexed: 28.76 mm/m²), with loss of sinotubular junction architecture. The proximal ascending aorta measured 70 mm (indexed: 31.96 mm/m²), confirming severe aneurysmal dilation.

**Figure 2:**
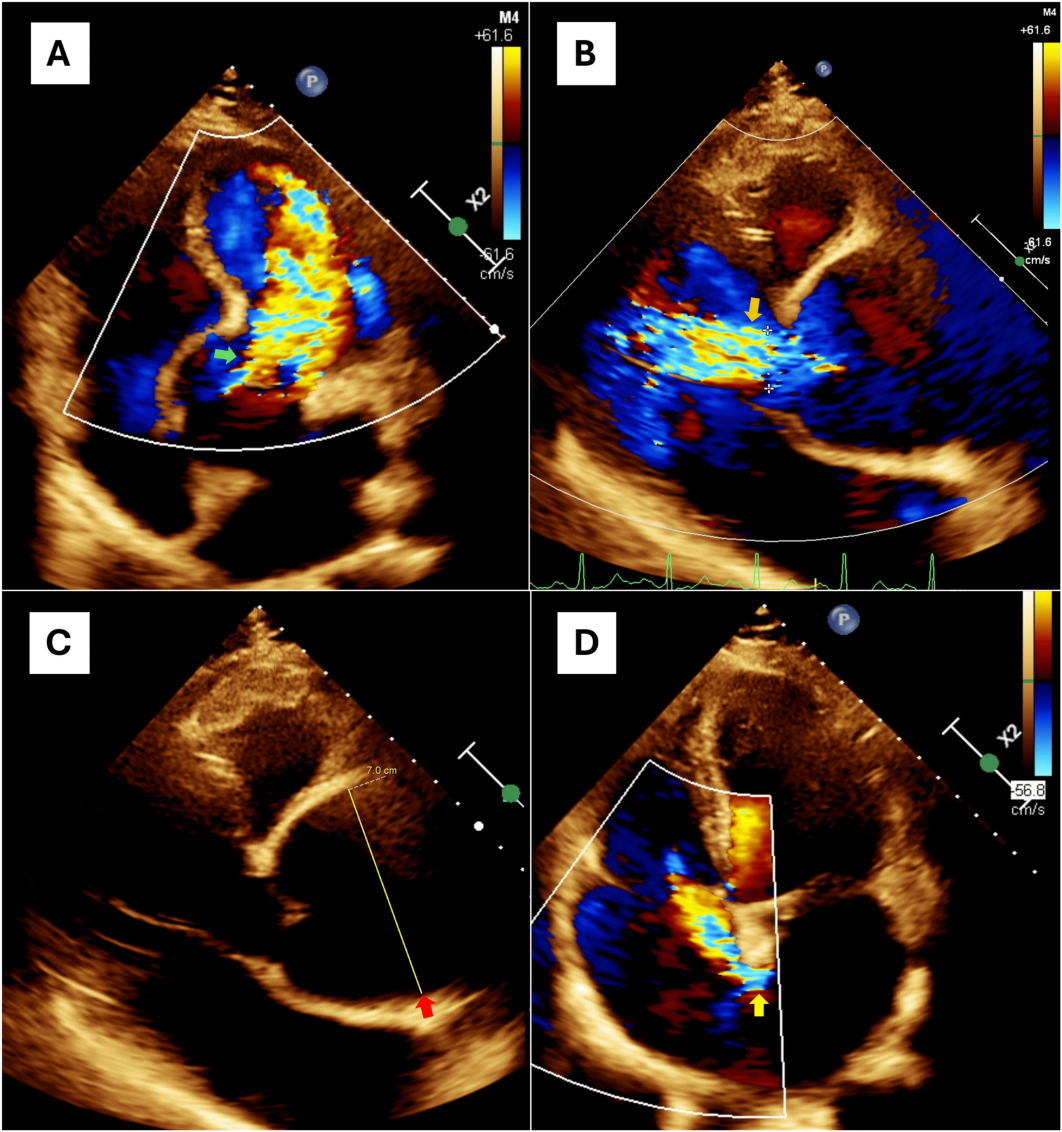
Transthoracic echocardiography findings A. Apical five-chamber view showing a severe aortic insufficiency jet (green arrow); B. Parasternal long-axis view demonstrating a holodiastolic aortic insufficiency jet (orange arrow); C. Aneurysmal dilation of the aortic root and loss of the sinotubular junction (red arrow); D. Apical four-chamber view, color Doppler imaging confirming a non-restrictive left-to-right shunt across the 11 mm atrial septal defect (yellow arrow).

Concomitantly, TTE documented severe aortic regurgitation characterized by a central jet with a width of 18 mm, occupying 68% of the left ventricular outflow tract (Figure 2). Additional severity markers included a vena contracta of 0.8 cm, a short pressure half-time of 168 ms, and the presence of holodiastolic retrograde flow in the descending aorta.

The left ventricle (LV) demonstrated generalized hypokinesia with a reduced ejection fraction (LVEF) of 40%. Evidence of LV remodeling was documented by an increased left ventricular end-diastolic diameter of 62 mm (reference range (RR): 42-58 mm) and a left ventricular end-systolic diameter of 51 mm (RR: 25-40 mm). Furthermore, the left ventricular end-systolic volume index was elevated at 43 mL/m² (RR: 11-31 mL/m²), confirming chamber dilation.

Incidentally, an 11 mm ostium secundum atrial septal defect (ASD) with a left-to-right shunt was detected (Figure 2). This combination contributed to biventricular remodeling; the right ventricle (RV) showed a basal diameter of 43 mm, a mid-diameter of 43 mm, and a longitudinal length of 80 mm, although RV function remained preserved (tricuspid annular plane systolic excursion 18 mm, S' 9.7 cm/s, and fractional area change 53%).

**Figure 3:**
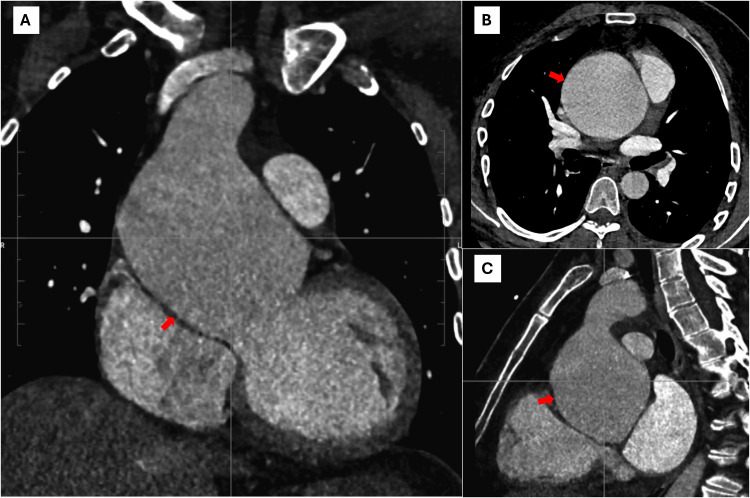
Computed tomography angiography of the aorta findings A. Coronal reconstruction showing massive aneurysmal dilation of the aortic root and ascending aorta (red arrow). B. Axial slice demonstrating the maximum transverse diameter of the aortic aneurysm, with loss of normal relationship to adjacent structures (red arrow). C. Sagittal reconstruction illustrating the longitudinal extent of the ascending aortic dilation (red arrow).

To further describe the vascular anatomy, a CT angiography (CTA) was performed. It confirmed the large size and revealed a valvular plane measuring 38 x 34 mm, sinuses of Valsalva of 70 mm (indexed: 31.9 mm/m²), and a maximum ascending aortic diameter of 76 mm (indexed: 34.7 mm/m²). Notably, the CTA documented a complete loss of the sinotubular junction structure (Figure 3).

Given the imminent risk of rupture, the multidisciplinary heart team decided to perform radical surgical intervention. A Bentall-Bono procedure was performed using a mechanical valved conduit to replace the aortic root and reimplant the coronary arteries. Simultaneously, closure of the ASD was performed. The intraoperative findings confirmed an extremely thin aortic wall and notably friable atrial tissues, a hallmark of MFS. To address this and ensure a secure repair, the ASD was closed using a pericardial patch. This surgical reinforcement was essential to prevent suture pull-through or late dehiscence, providing a more stable and tension-free repair compared to primary suture closure in such compromised connective tissue. The patient had a satisfactory recovery and was discharged on the seventh postoperative day. He demonstrated a significant clinical improvement, moving from New York Heart Association (NYHA) class IV to NYHA class II [8]. Although a formal follow-up echocardiogram for a new LVEF quantification is pending, the resolution of congestion and the patient's ability to perform activities of daily living independently provide objective evidence of hemodynamic recovery.

## Discussion

The understanding of the pathophysiology of MFS has evolved from a purely structural perspective to one involving a complex dysregulation of cell signaling. Mutations in FBN1 not only weaken the microfibrils of the extracellular matrix but also disrupt the sequestration of TGF-β, promoting aberrant tissue remodeling in the aortic media [1,3,9]. In this patient, the Steinberg and Walker-Murdoch signs (Figure 1) represented an external manifestation of systemic laxity, which, internally, resulted in an aorta with critically compromised structural integrity [4]. By quantifying these findings using the revised Ghent nosology score (Table 1) [4], we validated the clinical diagnosis with a total of 8 points. This high degree of systemic involvement, even in the absence of pectus deformities, underscores the severity of the underlying connective tissue disorder and its correlation with the massive aortic dilation observed.

From a hemodynamic perspective, the case presented an unusual biventricular "double impact" phenomenon. While severe aortic regurgitation caused volume and pressure overload in the left ventricle, the ASD created a left-to-right shunt that overloaded the right heart chambers and pulmonary vasculature [10,11]. This association, although infrequent and poorly reported in the literature, dramatically worsens cardiac remodeling, explaining why a young patient developed an LVEF of 40% in a short period. The flow from the ASD can be masked by the turbulence of aortic regurgitation, underscoring the need for meticulous echocardiography to detect coexisting congenital anomalies [4,10].

Managing ASD in patients with connective tissue disorders presents unique dilemmas. Although percutaneous closure is the standard in the general population, in MFS, there is a well-founded concern about the fragility of the atrial tissue. FBN1 deficiency makes the atrial septum prone to distension; The literature has reported cases of tissue erosion or migration of the occluding device due to the tissue not providing a firm anchor [12]. In this scenario, primary surgical closure during aortic repair was the safest approach, eliminating the risk of late complications associated with percutaneous prostheses in deficient tissue [12,13].

Regarding the aortic intervention, the magnitude of the dilation and the loss of the sinotubular junction necessitated a pragmatic decision [14]. Although valve-preserving techniques are attractive for avoiding permanent anticoagulation, the quality of the valve tissue in aneurysms of this size is often severely degraded. The Bentall-Bono procedure ensured definitive mechanical stability in a heart with prior ventricular remodeling [14,15]. Current guidelines reinforce that, in patients with diameters greater than 50 mm, radical surgery is the cornerstone for preventing catastrophic dissection [5,16].

This case report has inherent limitations. As a single-patient study (n=1), the successful outcome of combining a Bentall-Bono procedure with primary ASD closure cannot be generalized to all MFS patients with similar comorbidities. A significant limitation was the inability to perform TEE or FGD-PET due to patient intolerance and resource constraints, which could have provided a more definitive exclusion of infective endocarditis. Furthermore, the lack of a long-term follow-up beyond six months and the absence of a control postoperative echocardiogram to quantify the recovery of LVEF represent follow-up biases. To overcome these limitations, prospective registries of MFS patients with rare congenital associations are needed to establish standardized surgical protocols. Additionally, ensuring a multidisciplinary heart team approach and long-term longitudinal monitoring with standardized imaging remain the best strategies to mitigate individual case bias and improve clinical outcomes.

Finally, this case highlights the value of a comprehensive clinical approach. The successful resolution depended not only on technical skill but also on a holistic vision that integrated the stabilization of septic shock, the recognition of physical findings, and surgical planning that addressed two critical pathologies in a single procedure [17,18]. This strategy not only averted a life-threatening aortic rupture but also facilitated the restoration of functional capacity, offering the prospect of a high-quality life to a young patient facing a complex systemic disease.

## Conclusions

MFS requires a multimodal diagnostic approach and individualized treatment. The association of a severe aortic aneurysm, severe aortic regurgitation, and a hemodynamically significant ASD creates a critical biventricular challenge that must be addressed through concurrent surgical intervention. In patients presenting with sepsis and valvular disease, a rigorous exclusion of IE using the modified Duke criteria is mandatory before proceeding with radical surgery. This case underscores that surgical correction, incorporating techniques such as pericardial patch closure to address extreme tissue fragility, is the most effective strategy for modifying the natural history of the disease. Ultimately, a thorough physical examination and multidisciplinary planning remain fundamental to ensuring long-term survival and restoring functional capacity in young individuals with complex connective tissue disorders.

## References

[1] Judge DP, Dietz HC (2005). Marfan's syndrome. Lancet.

[2] Dietz HC, Cutting GR, Pyeritz RE (1991). Marfan syndrome caused by a recurrent de novo missense mutation in the fibrillin gene. Nature.

[3] Zeigler SM, Sloan B, Jones JA (2021). Pathophysiology and pathogenesis of Marfan syndrome. Adv Exp Med Biol.

[4] Loeys BL, Dietz HC, Braverman AC (2010). The revised Ghent nosology for the Marfan syndrome. J Med Genet.

[5] Mazzolai L, Teixido-Tura G, Lanzi S (2024). 2024 ESC Guidelines for the management of peripheral arterial and aortic diseases. Eur Heart J.

[6] Milewicz DM, Braverman AC, De Backer J (2021). Marfan syndrome. Nat Rev Dis Primers.

[7] Li JS, Sexton DJ, Mick N (2000). Proposed modifications to the Duke criteria for the diagnosis of infective endocarditis. Clin Infect Dis.

[8] Fisher JD (1972). New York Heart Association Classification. Arch Intern Med.

[9] Neptune ER, Frischmeyer PA, Arking DE (2003). Dysregulation of TGF-beta activation contributes to pathogenesis in Marfan syndrome. Nat Genet.

[10] Agrawal PK, Dev S, Panda SK, Yadav SS, Srivastava A (2025). Beyond the usual spectrum: Atrial septal defect in a Marfan syndrome patient with severe aortic pathologies. J Family Med Prim Care.

[11] Munde K, Agrawal PK, Yadav SS (2025). Marfan's syndrome with atrial septal defect rare clinical association. Int J Card Res.

[12] Day CM, Mulla N, Martens T (2021). The ASD that wouldn't go away: an unusual case of ASD device failure in a patient with Marfan syndrome. Congenit Heart Dis.

[13] Isselbacher EM, Preventza O, Hamilton Black J 3rd (2022). 2022 ACC/AHA Guideline for the Diagnosis and Management of Aortic Disease: A Report of the American Heart Association/American College of Cardiology Joint Committee on Clinical Practice Guidelines. Circulation.

[14] Woldendorp K, Starra E, Seco M (2014). Replacement of the aortic root with a composite valve-graft conduit: risk factor analysis in 246 consecutive patients. Heart Lung Circ.

[15] Coselli JS, Volguina IV, Nguyen L, Green SY, LeMaire SA, Moon MR (2023). Outcomes of aortic root replacement in patients with Marfan syndrome: the role of valve-sparing and valve-replacing approaches. Ann Cardiothorac Surg.

[16] Erbel R, Aboyans V, Boileau C (2014). 2014 ESC Guidelines on the diagnosis and treatment of aortic diseases: Document covering acute and chronic aortic diseases of the thoracic and abdominal aorta of the adult. The Task Force for the Diagnosis and Treatment of Aortic Diseases of the European Society of Cardiology (ESC). Eur Heart J.

[17] Dietz HC, Reed E. (2014). Marfan Syndrome. The Online Metabolic and Molecular Bases of Inherited Disease. Valle DL, Antonarakis S, Ballabio A, et al..

[18] Vahanian A, Beyersdorf F, Praz F (2022). 2021 ESC/EACTS Guidelines for the management of valvular heart disease. Eur Heart J.

